# Metabolomics as a Tool to Investigate Abiotic Stress Tolerance in Plants

**DOI:** 10.3390/ijms14034885

**Published:** 2013-03-01

**Authors:** Vicent Arbona, Matías Manzi, Carlos de Ollas, Aurelio Gómez-Cadenas

**Affiliations:** Plant Ecophysiology and Biotechnology Laboratory, Departament of Agricultural and Environmental Sciences, Universitat Jaume I, Castello de la Plana E-12071, Spain; E-Mails: arbona@uji.es (V.A.); mmanzif@gmail.com (M.M.); deollas@uji.es (C.O.)

**Keywords:** cold, heat, metabolite profiling, mQTL, omics, osmoprotectants, oxidative stress, salt stress, soil flooding, water stress

## Abstract

Metabolites reflect the integration of gene expression, protein interaction and other different regulatory processes and are therefore closer to the phenotype than mRNA transcripts or proteins alone. Amongst all *–omics* technologies, metabolomics is the most transversal and can be applied to different organisms with little or no modifications. It has been successfully applied to the study of molecular phenotypes of plants in response to abiotic stress in order to find particular patterns associated to stress tolerance. These studies have highlighted the essential involvement of primary metabolites: sugars, amino acids and Krebs cycle intermediates as direct markers of photosynthetic dysfunction as well as effectors of osmotic readjustment. On the contrary, secondary metabolites are more specific of genera and species and respond to particular stress conditions as antioxidants, Reactive Oxygen Species (ROS) scavengers, coenzymes, UV and excess radiation screen and also as regulatory molecules. In addition, the induction of secondary metabolites by several abiotic stress conditions could also be an effective mechanism of cross-protection against biotic threats, providing a link between abiotic and biotic stress responses. Moreover, the presence/absence and relative accumulation of certain metabolites along with gene expression data provides accurate markers (mQTL or MWAS) for tolerant crop selection in breeding programs.

## 1. Metabolomics within the Context of Systems Biology

The phenotype of an organism is the result of the combination of multiple intertwined, dynamic and linear/non-linear interactions among different elements (DNA, RNA, proteins and metabolites) with the environment (developmental stages and/or adverse conditions such as salinity, temperature and water or nutrient availability). For this reason, most genome-scale studies require an accurate phenotype description besides the analysis of RNA transcripts, proteins and metabolites. Nevertheless, the sum of these three aspects does not provide a clear picture of the actual phenotype of a given organism but a sequential characterization of the elements one by one. This approach lacks the emerging properties that characterize biological organisms; therefore there is an increasing need for the integration of all these aspects [1–3]. This is of especial relevance when the objective is to understand how plants respond to environmental cues. In this sense, whereas gene and protein expression represent the *potential* of plants to respond to adverse conditions, metabolites constitute the true *integration* of these two aspects plus the influence of the environment and/or other organisms. However, we first need to understand what information can be extracted from the application of the different profiling (*omics*) methodologies and how can metabolomics help to better comprehend the nature of phenotypes. In addition, the physiological and biochemical effects of different abiotic stress conditions and how metabolite markers can be used for the selection of cultivars and/or rootstocks with improved yield/abiotic stress tolerance will be reviewed.

### 1.1. Omics Technologies: Transcriptomics, Proteomics and Metabolomics

In recent years, after the publication of *Arabidopsis* and Human genomes [4], a number of strategies have been developed to cover the entire three aspects or an organism’s biology, namely transcriptomics, proteomics and metabolomics. These technologies generate enormous amounts of information which has boosted up the field of bioinformatics, with thousands of new algorithms and software published every year. The development of these tools has allowed the inference of the causal correlation among the analyzed elements. Another important technological innovation has been the improvement in data storage as well as computational capacities involved in the acquisition and processing of large datasets. In addition, several web and software platforms aimed to share, integrate and visualize in a biological context the overwhelming amount of data have been developed.

The analysis of gene transcripts is probably the most developed field. Indeed, there are several platforms available: the extremely accurate qRT-PCR that allows only a limited number of genes to be analyzed [5]; the gene microarray technology allowing the analysis of thousands of genes at a time [6] and RNA Seq oriented to the performance of unbiased analysis of RNA transcripts [7,8] which generates gigabyte size readouts with all the RNA transcripts of a given cell, organism or tissue. The increasing amount of data generated pushed researchers to create databases of experiments covering different organs [9], tissues and cell types [10], developmental events [11] and environmental cues [12], allowing to take a sneak peek into the expression of gene candidates in advance. For example, Genevestigator [13] database allows a preliminary survey of the gene candidates before programming any experiment and *Arabidopsis* eFP Browser [14] shows gene expression at the organ level. Moreover, with RNA Seq is also possible to detect diverse variants of mRNA and other RNA molecules such as noncoding RNA or small RNAs in different organisms [15].

Following transcript analyses, proteins are the second most important aspect in defining an organism’s phenotype, as the product of gene expression. To cover up all the translational events from mRNA to functional proteins, analytical platforms necessarily need to be able to evaluate not only the presence/absence of a given protein but the potential post-translational modifications (e.g., phosphorilations, glycosilations or prenylations) and also the potential to assess protein-protein interactions. Available platforms include the traditional 2D gel electrophoresis (combined or not with fluorescent dyes, as in the DIGE technique, [16]) which is useful for protein fingerprinting when coupled to mass spectrometry (MS) for protein identification and, finally, the shotgun proteomics approach, based on nano-liquid chromatography (nanoLC) separation of matrices and MS detection, which offers a deeper and less biased coverage of the proteome including low abundant proteins [17]. The future challenges in proteomics are the development of new analytical techniques and workflows to overcome the lack of reproducibility and the implementation of new features for data exportation and comparison in databases [18].

The term metabolomics has been defined as the identification and quantitation of all low molecular weight metabolites in a given organism, at a given developmental stage and in a given organ, tissue or cell type [19,20]. This is a challenging task due to the wide array of molecules with different structures and chemical properties. For instance, it is estimated that a single accession of *Arabidopsis* contains more than 5000 metabolites, most of them yet uncharacterized. Unlike transcriptomics, there is no single approach to detect all compounds and the adequate combination of extraction and detection techniques is key to increase the coverage of the technique [21]. The most popular metabolomics techniques focus on metabolites with similar and specific chemical properties and are globally known as metabolite profiling only covering up a fraction of the metabolome. To achieve a comprehensive coverage of the vast range of metabolites present in the plant kingdom several analytical techniques consisting of a separation technique coupled to a detection device (usually MS) are combined. However, there are alternatives that dismiss the use of a separation technique such is the case of flow injection analysis coupled to MS (FIA/MS) or use different analyzers such as nuclear magnetic resonance (NMR) or Fourier Transform Infrared spectroscopy (FTIR) that are used only for fingerprinting purposes. The separation part provides the selectivity needed for certain groups of metabolites. For instance, gas chromatography (GC) is mainly intended for volatiles and primary metabolites (e.g., sugars, aminoacids or tricarboxylic acid (TCA) cycle intermediates) after derivatization [22]. On the other hand, although LC is very flexible and can be adapted to a vast array of compounds, it has been mainly used for secondary metabolites without prior derivatization [19]. In this sense, capillary electrophoresis (CZE) provides similar characteristics as LC but with the advantage that ionic metabolites can also be properly separated [23]. Among all analyzers that can be used with the separation techniques mentioned, the most popular in metabolomics are MS analyzers and, particularly, those providing accurate mass measures such as hybrid quadrupole/time-of-flight or orbitraps [19,24–26]. However, more targeted techniques are still extensively used for the quantitation of several plant metabolites and hormones due to their enhanced sensitivity and specificity [27–29].

As mentioned above, a serious drawback is the handling of the great amount of data generated. In addition, metabolites need to be properly annotated to obtain consistent and useful results. Whereas, for primary metabolites, it is much facilitated due to the availability of several public libraries for GC/MS studies such as [30,31], it is still a challenging task in the case of secondary metabolites, since no comprehensive database exists up to date [32–34]. Therefore, a future objective to achieve in these techniques is the standardization and annotation of data from multiple metabolomics technologies in public databases [35]. The future challenge is the integration of all three aspects within a single framework that will allow a better understanding of how plants respond to a changing environment.

### 1.2. Data Integration: Gene-Protein-Metabolite

The data collected from transcriptomics, proteomics and metabolomics needs to be combined to achieve a better understanding of the plant as a system. Several research groups have provided workflows to integrate all this information into a single pipeline.

#### 1.2.1. Transcriptomic-Proteomic

A recurrent topic in transcriptomics and proteomics is the correlation between the expression of protein-coding genes and the abundance of the corresponding proteins. There are studies that reported a moderate correlation (a Pearson’s correlation index of 0.4) between RNA and protein in unicellular organisms in steady-state conditions, increasing in stressed conditions (up to 0.7). However, this correlation has been shown to be lower in multicellular eukaryotes, indicating a major role of post-translational regulation in the activity of the cell [36]. Hence, the best functional insight can be obtained by combining measurements across technologies, and searching for broader groups of genes, proteins, and metabolites with regulatory relationships [37]. However, the extreme complexity of the underlying processes (such as the existence of yet unknown regulatory mechanisms) makes this a challenging task, in addition to several technical cross-platform issues [36].

#### 1.2.2. Transcriptomic-Metabolomic

Another possibility is to integrate transcription with metabolites. This integration can also help to unveil genes and processes underlying complex traits [38]. In order to facilitate the integration, several software packages have been developed such as MapMan [39] or, more recently, MetGenMap [40]. These computer programs have been successfully applied to identification of genes and metabolic pathways involved in germination, diurnal cycles [41] and seed dormancy [42]. Indeed, they have proven to be useful tools to predict the function of co-regulated genes under given conditions and to identify genes involved in metabolite biosynthesis and transcriptional regulation [43,44].

#### 1.2.3. Metabolomic-Proteomic

In non-targeted metabolomics, principal components analysis (PCA) and independent components analysis (ICA) are methods commonly used to perform pattern recognition. In addition, it is possible to strengthen this technique by including additional parameters such as external perturbations (stress), protein concentration, and/or enzyme activities, thus generating metabolite correlation networks. In line with this, Weckwerth and collaborators [45] exploited the improvements of ICA respect to PCA using an integrated metabolite-protein data matrix to separate the principal components of genotype (*Arabidopsis* WT *vs. Arabidopsis* phosphoglucomutase mutant) throughout a diurnal rhythm. Using a similar approach, the starch and raffinose metabolisms in response to low and high temperature have been recently dissected with an integrative approach in *Arabidopsis thaliana*[46].

Overall, transcript profiling is the most mature technique in the systems biology field, allowing acquisition of exhaustive and large-scale datasets. In addition, there are several public databases where annotation of candidates is performed automatically. In the case of proteomics and metabolomics, great technical challenges exist to overcome the same large-scale coverage of the different transcriptomics platforms due to the diverse chemical nature of both proteins and metabolites and the impossibility of pre-amplification as in the case of nucleic acids, making instrument sensitivity a serious challenge. Nevertheless, specific databases with well annotated data begin to spread and the common effort is beginning to give promising results: VirtualPlant [47] and GeneMANIA [48] allow the combination of different large-scale data to start modeling the complex behavior of organisms.

## 2. Abiotic Stress, Causes and Physiological Responses

Physiological responses of plants to environmental cues involve changes not only at the transcriptional level but also in post-translational protein modifications and metabolite alteration and/or accumulation, leading to a particular physiological response or phenotype [49]. Plant physiological responses to stress are oriented towards tolerance, sensitivity or avoidance of the stressful conditions [50]. In the natural environment, adverse situations are always a combination of several stress factors (e.g., water limitation, high temperature or irradiation and high osmolality). This is the reason why it is always difficult to determine which stress factor (if not all) is behind the elicitation of a particular physiological response.

To simplify the effect of environmental conditions on physiological responses, researchers have traditionally subjected plants to a specific stress factors under highly controlled conditions and keeping the rest of parameters at optimum values, thus neglecting their contribution to the physiological responses [51]. In this review we will follow primarily this approach although additional comments on combined-stress experiments will be also provided.

### 2.1. Drought

The most important stress factors limiting plant growth, reproductive development and, ultimately survival, is drought. This stress factor is related to water supply limitation, not only understood as the strict cease in water supply but also as continuous water deficit throughout growth, reproductive or developmental stages [52]. One of the most important physiological parameters being affected by drought or water shortage is photosynthesis; in this sense both water and salt stress are quite similar causing a progressive and severe reduction in the CO_2_ assimilation capacity. This decrease in net photosynthetic rate is first associated to a stomatal closure induced by a decline in leaf cell turgor that limits diffusion of CO_2_ into the substomatal chamber. Under these conditions that diminish CO_2_ diffusion through the mesophyll, photoinhibition, a process that reduces quantum yield of PSII and induces photorespiration and H_2_O_2_ production [53] is likely to occur. Hence, the production of Reactive Oxygen Species (ROS) is one of the primary responses to stress following the decline in photosynthesis, causing cell damage but also a signal to be transmitted [54,55]. Massive ROS production, if not controlled by antioxidant mechanism, can induce photosynthetic pigment bleaching, thylakoid membrane degradation and alteration of protein structure and function. Plants respond to the induced oxidative stress by overproducing antioxidant compounds such as ascorbic acid, glutathione and polyphenols [56,57]. Besides this, drought stress induces as a general response the accumulation of several aminoacids such as valine, leucine, isoleucine and agmatine (as a precursor of polyamines) along with carbohydrates and carbohydrate alcohols which, in combination with proline (Pro), could have an osmoprotective role. In addition, while increases in carbohydrates and their alcohols occurred as a short term response and likely not under abscisic acid (ABA)-dependent signaling, the accumulation of Pro and other aminoacids was observed after long term drought and seemed to be under ABA regulation [58].

### 2.2. Salinity

Another major factor limiting plant growth and production is salinity. This stress factor is derived from the massive accumulation of salts near the root zone and causes an osmotic effect followed by a specific toxicity, derived from the accumulation of saline ions in plant tissues [59]. The most studied effect is the salinity associated to the accumulation of NaCl due to overexploitation of freshwater resources and the subsequent marine intrusion, known as primary salinization [60]. In natural environments, osmotic and ionic effects co-occur and usually the symptoms of ion toxicity precede leaf drop [59]. Under these conditions, non-tolerant plants exhibit succulence, arrest in growth and reproductive development, continuous organ abscission and, if the saline conditions persist, death [59]. However, under artificial stress conditions, plants are suddenly exposed to high saline concentrations (e.g., 100 or 200 mM NaCl) or in increasing steps (25, 50, 75, 100 mM NaCl). This triggers the massive accumulation of saline ions and compatible osmolyte biosynthesis to counterbalance the severe osmotic effect [59].

### 2.3. Soil Flooding

Water stress is either associated to a deficit in water availability or to an excess irrigation that impairs water uptake. In particular, soil waterlogging constitutes a seasonal stress factor whose incidence on crops is difficult to predict. When the soil water content rises above field capacity a fast depletion of O_2_ occurs due to the low diffusion rate of this gas in water together with the consumption made by plants roots. This O_2_ depletion can occur in less than 24 h, depending on the root/microbiota biomass present in soil [61–63]. In citrus, soil flooding causes a progressive reduction in gas exchange parameters that is proportional to the relative tolerance of the different genotypes. Indeed, tolerant genotypes maintain CO_2_ assimilation rate and carboxylative efficiency at control levels for longer time than sensitive genotypes under continuously flooded conditions [63]. Thus, tolerance is linked to the ability of maintaining gas exchange parameters which is, in turn, related to transpiration and, ultimately, to plant vigor.

### 2.4. Temperature Stress

In a climate change context, the effect of high temperatures has been recently reviewed by Mittler and co-workers [64]. In general, heat stress affects the stability of proteins, nucleic acids, the cytoskeleton structure and the efficiency of enzymatic reactions, causing a severe metabolic imbalance. The sensing of heat stress takes place at the plasma membrane of cells which is physically altered, acting as a real thermometer [65]. Heat also causes several metabolic changes associated to impairment in electron transport chains and production of ROS such as the membrane bound NADPH oxidase [64]. In addition, another primary target of this stress is the photosynthetic system, especially the PSII and the oxygen-evolving complex, the ATP generating system and the carbon assimilation process [65].

In the opposite, the effect of low temperatures above freezing (0–15 °C) is also an important stress factor limiting crop productivity. As in heat stress, photosynthesis is largely affected by cold stress. The cessation of growth resulting from cold stress reduces the capacity for energy utilization, causing a feedback inhibition of photosynthesis and production of ROS. Membrane composition and fluidity is the key change involved in low temperature perception. Indeed, at low temperatures cell plasma membranes undergo phase transition in which fluidity of membrane is reduced to form a solid gel [66], which is used by plant cells to sense cold stress.

## 3. Effect of Abiotic Stress on Plant Biochemistry: Metabolites as Effectors of Tolerance/Damage and Genes Involved

In recent years, metabolomics techniques have drawn attention of researchers in different areas of plant science such as phytopathology, botany and systematics, stress and environmental physiology and, of course, phytochemistry. In general terms, metabolomics is deeply related to phytochemistry or natural products chemistry in the same way as genomics is rooted in classical molecular biology (one-gene-at-a-time). As a step forward, the aim of modern metabolomics is the identification and quantitation of all metabolites in a given plant species at a given developmental stage under particular environmental conditions [63]. However, as indicated above, this is not possible up-to-date since the immense chemical diversity of plant metabolites cannot be unraveled with a single analytical technique [67,68]. Plant metabolomics have been used for several purposes: (1) evaluation of the impact of stress/treatment on plant metabolism, (2) tracking of a certain compound or compound category within a particular biosynthetic/degradation pathway and (3) classification of samples [1]. Depending on the pursued objective, a targeted or a non-targeted approach should be chosen. Since both the extraction and the analytical technique chosen might influence the array of compounds analyzed in a metabolomics platform, it can be accurately stated that almost every current metabolomics platform is indeed a targeted technique.

As a whole, the actual metabolite composition of a given plant species is the result of a particular gene expression profile. When a certain metabolic pathway is activated, precursors and intermediates are channeled to produce a bioactive molecule: an antioxidant, a signaling compound, a cell structure biosynthesis intermediate or even a storage compound. The production of these compounds can be regulated in turn by other compounds (signaling molecules, such as plant hormones) not related to the regulated pathway or intermediates that can feedback activate or inactivate different metabolic steps. In addition, in pathological conditions, metabolites can also constitute cell damage subproducts such as malondialdehyde (MDA), lipid peroxides and DNA fragments resulting from oxidative or enzymatic cleavage. Considering the metabolome, the balance between defense, signaling and damage metabolites can be used to assess plant tolerance to a certain stress situation [69,70].

The most sensitive mechanism to abiotic stress is photosynthesis and when plants are subjected to adverse environmental conditions such as drought, salinity, heat or cold, to name a few, carbon assimilation and the primary metabolism are largely affected. Among all primary metabolites: sugars, sugar alcohols and aminoacids are the most important metabolites which concentration in plant tissues is affected by stress, usually as a downstream result of an impairment in the CO_2_ assimilation process, but also as a result of a complex regulatory network [71,72]. Nevertheless, due to the great differences in concentration (usually several orders of magnitude) changes in secondary metabolites levels cannot be simply inferred from variations in their primary metabolite precursors and is usually a result of a complex regulatory process. For this reason, stress-associated changes in secondary metabolites will be considered and reviewed separately.

### 3.1. Primary Metabolism and Osmoprotectants

#### 3.1.1. Carbohydrates

Carbohydrate metabolism plays an important role in the stress tolerance conditions as it is directly linked to photosynthetic performance. During the stress period, plants use starch and fructans as a source of energy instead of glucose [73] as evidenced by the increase in β-amylase activity [74]. These simple sugars can act as osmolytes maintaining cell turgor, stabilizing cell membranes and preventing protein degradation [75]. Indeed, under water deficit the concentration of soluble carbohydrates such as glucose and fructose increases in roots of stressed plants [76] whereas sucrose is transported to the root tips promoting growth and contributing to the increase in root-to-shoot ratio [77]. Moreover, high amounts of non-reducing disaccharides such as trehalose can accumulate in tolerant plants subjected to desiccation. Nevertheless, although trehalose-overaccumulating transgenic plants displayed an enhanced stress tolerance; no increase in trehalose content was observed, excluding a direct role of this metabolite in stress protection [78]. Other sugars with no energetic role, such as the oligosaccharides raffinose and stachyose accumulate in different plant species in response to a broad range of abiotic stress conditions such as drought, salinity or extreme temperatures [73]. These compounds have been associated to a reduction in oxidative membrane damage and ROS scavenging [79]. Polyols are also implicated in stress tolerance due to its action as scavengers of hydroxyl radicals. In addition, accumulation of sugar alcohols like mannitol or sorbitol has been linked to stress tolerance [80].

#### 3.1.2. Aminoacids: Proline

**Figure 1:**
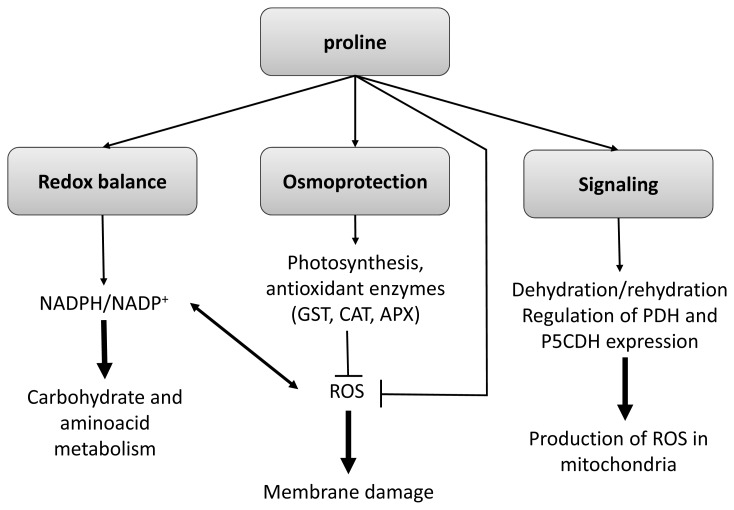
Multifaceted role of Proline in the responses of plants to stress. Abbreviations in the figure, GST: glutathione *S*-transferase, CAT: catalase, APX: ascorbate peroxidase, PDH: proline deshydrogenase and P5CDH: pyrroline 5′-carboxylate deshydrogenase. Adapted from [82].

During abiotic stress conditions, plants induce the synthesis of osmolytes such as soluble sugars and amino acids which contribute to turgor maintainance by osmotic adjustment [81,82]. Among amino acids, Pro is the main effector in this response (in addition to hexoses), contributing to around 50% of the osmotic adjustment in maize root tips [77]. Indeed, increases in Pro content have been reported in response to different abiotic stress conditions like salt stress [83,84], soil flooding [85], drought [81] or extreme temperatures [66,73]. However, whether Pro can counteract and protect against abiotic stress or not is still a question of debate. The biosynthesis of Pro is activated under dehydration whereas rehydration induces the opposite pathway (Figure 1); the target enzyme is a pyrroline-5-caboxylate synthetase (P5CS) located mainly in cytoplasm [82]. For many years, the ability to synthesize and accumulate Pro has been considered a tolerance trait as P5CS gene expression has been reported to be highly correlated with drought stress and the accumulation of Pro [86]; however, in response to different abiotic stress conditions, overaccumulation of Pro in leaves of several citrus genotypes and model plants was associated to sensitivity [85,87]. Nevertheless, transformation of citrus with a P5CS gene under the control of Cauliflower mosaic virus 35S rRNA promoter led to an increased tolerance to drought and an improved ability for osmotic adjustment [81,88].

Besides the known activity of Pro as a compatible solute several researchers have also claimed its role in ROS scavenging [81] and DNA, membrane and protein stabilization [82]. However, greater increase in Pro levels does not always result in alleviation of oxidative damage [83,85]. Recently, it has been suggested that Pro overaccumulation could increase ROS and MDA production probably via pyrroline-5-carboxylate and by inhibition of ABA and ethylene biosynthesis resulting in a decrease in stress tolerance [89].

#### 3.1.3. Polyamines

Polyamines (PA) are nitrogenous aliphatic molecules of low molecular weight and positively charged which are present in most living organisms. Several regulatory, protective and ROS scavenging roles have been assigned to these molecules related to the aminoacid metabolism [90]. Several abiotic stress conditions induce PA accumulation which has been positively correlated with stress tolerance [91,92]. The most common PAs found in higher plants are putrescine (Put), spermidine (Spd) and spermine (Spm) and can be present as free and conjugated forms. Indeed, PA conjugation as hydroxycinnamic acid amides such as coumaroylputrescine, feruloylputrescine, dicoumaroylspermidine, diferuloylspermidine or diferuloylspermine contributes to regulate free PAs levels in plants [93]. As a whole, concentration of free PAs are tightly controlled by balancing biosynthesis, catabolism and conjugation, which is especially relevant during adverse environmental conditions [94]. The specific way by which these compounds increase stress tolerance in plants still remains unknown. Indeed, their role as compatible solutes has been recently questioned based on its lower concentration in comparison to a classical osmolyte such as Pro [95]. Exogenous application of PA to plants subjected to drought alleviated stress pressure by reducing H_2_O_2_ and MDA levels through the increase in peroxidase and catalase enzyme activity and Pro levels [96,97]. Moreover, Put levels during stress conditions were positively correlated with reduced levels of H_2_O_2_ and lipid peroxidation and increased antioxidant enzyme activity and carotenoid concentration [98]. This improved stress tolerance in plants with high Put levels was correlated with a reduced stomatal aperture and lower transpiration rate [98]. To this respect, it has been suggested a positive feedback mechanism between Put and ABA, indicating an effect on stomatal opening through ABA signaling [99]. Other PAs such as Spm and Spd, have been associated to the induction of nitric oxide (NO) which is involved in signaling under abiotic stress conditions [100]. However, there is no clear agreement about the specific role and implication of each PAs in stress tolerance. This could be due to a different contribution of PAs in each specie and stress condition [95,101] or to the induction of a different set of genes involved in responses to abiotic stress by exogenous application of every PA.

#### 3.1.4. Integration of Metabolites as Physiological Effectors

In general terms, it is difficult to assign a protective role to a certain metabolite since no direct relationship has been demonstrated for most of the primary metabolites described. To this regard, one approach to identify adaptive metabolic changes is the comparison between stress-adapted *versus* non-adapted species or cultivars [102]. When using this approach in crop plants, such as the forage legume *Lotus corniculatus*, a low degree of overlapping in drought-elicited metabolic responses was found among closely related species [103]. This could indicate a high degree of exchangeability between small molecular weight metabolites in terms of biological function. In response to salt stress, plants of the *Lotus* genus exhibited a similar metabolic response regardless their tolerance, showing a general increase in shoot aminoacid concentration (including Pro) and a downregulation of Krebs cycle intermediates [104]. However, although with similar tendency, the degree of alteration was different between glycophyte and halophyte species. These differences could be associated to different basal levels, which are in agreement with a pre-adaptation model [87,105]. In maize, a NMR-based metabolite profiling study confirmed that early effects of salt stress are related to the osmotic component of salinity. In addition, results were consistent with an osmotic effect stronger in shoots than in roots [106]. However, these studies have the limitation of not proving the causal relationship between specific metabolic changes and stress tolerance and, moreover, do not allow the identification of the underlying molecular mechanisms [102].

It has been recently reported that, in non-adapted *Thellungiella* accessions, sugars and polyamines could be involved in the mechanisms of cold adaptation [107]. Similar metabolite fingerprints were found in acclimation of *Drosophila melanogaster* individuals to cold conditions [108] indicating that the mechanisms to cold adaptation could be the same among kingdoms. In a recent review, Janská and co-workers summarized all the important metabolic changes occurring in cold acclimation, reinforcing the idea that the synthesis of cryoprotectant molecules is of vital importance [109]. Among these cryoprotectans, sugars, sugar alcohols and low molecular weight nitrogenous compounds such as Pro and glycine betaine were shown to be most important. Hence, the accumulation of these molecules in adapted individuals could contribute to a higher cold stress tolerance [110]. Similarly, acclimation of plants to heat stress involves the accumulation of sugars such as maltose, sucrose and trehalose, aminoacids such as α-alanine and sugar alcohols such as glycerol. Non-targeted metabolomic studies revealed an effect on pantothenate/CoA pathways that could not be otherwise found [111].

It is important to note that there is a clear difference in phenotypic and metabolic responses between field and greenhouse grown plants. In a recent work, samples from maize hybrids differing in drought tolerance and subjected to dehydration under greenhouse conditions were analyzed by means of GC/MS. Phenotyping of the plants could not clearly differentiate between tolerant and susceptible genotypes. However, it was possible to confirm certain metabolite responses associated to tolerance already observed under field conditions [112], demonstrating the power of metabolite profiling techniques to show differences when phenotypes are masked by environmental factors. Nevertheless, it is important to highlight the importance of a proper phenotype evaluation in the assessment of stress tolerance and the development of selection markers (either genetic or metabolic) [52]. To this regard, the selection of markers for phenotype assessment is not trivial. For instance, when considering tolerance to salt stress, the ability to maintain growth under high salinity even with high Na^+^ leaf concentrations, was considered a tolerance trait in barley [113]. This is in contrast with salt stress mechanisms in other glycophytes, for instance, in citrus where the ability to reduce Cl^−^ uptake to the aerial part is considered a tolerance trait [114]. Moreover, in other stress situations such as heavy metal contamination, the ability to reduce metal uptake to the photosynthetic organs is considered a tolerance trait. In these species, phytochelatin biosynthesis and glutathione metabolism exhibit a remarkable upregulation when grown under high metal concentrations [115]. However, there are certain species known as hyperaccumulators that are able to overaccumulate metals. A direct correlation was found between citrate and metal accumulation in all species analyzed and particularly hyperaccumulators showed high concentrations of malonate in leaves, probably acting as a metal storage mechanism [116]. To add more complexity, other quantitative trait such as Fe deficiency tolerance is, on the contrary, evidenced by the higher ability to assimilate Fe from the substrate even when present at very low concentrations. In fruit crops such as citrus, responses to iron deficiency in susceptible genotypes have been associated to a decrease in the ability for ROS scavenging and the induction of genes involved in the biosynthesis and modification of cell wall components [117]. In pea plants, however, Fe-deficiency in tolerant pea cultivars, induced a strong accumulation in nitrogenous, sulphurous and Krebs cycle metabolites associated to N-recycling, increased glutathione and the production of metabolites involved in Fe sequestration, mainly citric acid, suggesting a strategy oriented towards the improvement of Fe uptake and the defense against the associated oxidative stress [118]. All these results point out that more physiological information is needed to understand how plants respond to abiotic stress, not to use physiological responses as stress tolerance markers, yet these are highly influenced by environmental conditions, but to use them to evaluate stress responses.

### 3.2. Secondary Metabolites: Antioxidants, Defense Compounds and Regulatory Metabolites

Plants can synthesize a vast array of “special” metabolites that do not seem to have any essential role in plant physiology. However, the occurrence of these compounds provides particular ecological advantages of certain species to colonize specific habitats. Among many others, phenolics and carotenoids provide protection against excess light and UV irradiation, glucosinolates and alkaloid glycosides are important feeding deterrents against herbivory and certain terpenoids can act as semiotic or signaling compounds. The array of secondary metabolites is specific to a plant species and their biosynthesis is tightly regulated by the developmental stage, tissue or cell group, and of course, by several stress situations [19,57,119].

#### 3.2.1. Phenolic Compounds

The compound class composed by phenolic metabolites constitutes the most diverse array of secondary metabolites found in plants and includes phenylpropanoids (cinnamic, coumaric, caffeic and ferulic acids) and its derivatives such as polyphenolics, namely flavonoids, anthocyanins and tannins. These compounds are synthesized through the shikimate pathway leading to phenylanaline which is the substrate of phenylalanine ammonia lyase (PAL) which is the key enzyme in the phenolic biosynthesis pathway. This enzyme catalyzes de deamination of phenylalanine rendering cinnamic acid, the first precursor of flavonoid and lignin biosynthesis. Under different adverse environmental conditions, the increase in PAL activity as well as other enzymes of the phenylpropanoid pathway has been reported [120]. These secondary metabolites are thought to play a role in the side effects derived from environmental changes, such as increase in insect predation. This is of especial relevance in a climate change context, in which it is expected that the ambient CO_2_ concentration rises considerably. In a recent publication, plants of *Brassica rapa* were subjected to increased CO_2_ (744 ppm, about 2-fold the current ambient levels) concentrations for more than 40 days [121]. Under these conditions, plants increased trichome density as well as the amount of constitutive phenolics. However, the ability to induce new secondary metabolites was partially impaired suggesting a negative effect on the ability to respond to herbivory damage. Another expected effect associated to global climate change is warming. According to a report in the Intergovernmental Panel on Climate Change, global mean temperature will rise up to 1 °C in the following 12 years, and to 3 °C in 80 years from now [122]. It is known that heat induces PAL activity and the production of phenolics and, at the same time, reduces their oxidation contributing to heat stress acclimation. In *Arabidopsis thaliana*, UV-B treatment increased the concentration of flavonol (naringenin, kaempferol and quercetin hexosides) and derivatives (cinnamoyl and coumaroyl) that may act as UV-B radiation screen; however co-treatment with the flagellin effector flg22 induced a partial shutdown of the pathway [123]. In another work, this treatment enhanced resistance to infection with *Botrytis cinerea* spores [124] indicating that the induced flavonols could have also a protective effect against biotic stressors. In response to soil flooding, more than 40 flavonoid in leaves of two citrus rootstock species differing in stress tolerance were identified [57]. After metabolite profiling analysis of samples from flooded and control plants, it was found that flavonoid levels were much decreased in the sensitive genotype, suggesting an efficient redox balance in the tolerant species. Phenylpropanoids are precursors of lignins, which constitute an important stress defense mechanism, especially at the root level where can modulate cell wall composition and stiffness [125,126].

Other phenylpropanoids derived from the isochorismate pathway collectively known as benzenoids are found as volatile forms, esterified to other secondary metabolites or bound to cell walls [127]. Among the volatile forms methyl salicylate [128] and methyl benzoate have an important activity in plant defense from pathogens [129]. Another well-known benzenoid is salicylic acid (SA), a plant hormone that has been traditionally involved in pathogen defense [130] but has been proved effective in alleviating the damage induced by several abiotic stress conditions [131]. Mechanistically, SA may induce little bursts of H_2_O_2_ production resulting in mild oxidative stress which, in turn, could enhance the antioxidant activity, improving stress tolerance [132].

#### 3.2.2. Glucosinolates

Glucosinolates are nitrogen and sulphur-containing compounds derived from aminoacids such as methionine, alanine, valine or leucine (aliphatic); phenylalanine or tyrosine (aromatic) and tryptophan (indolic glucosinolates). This class of compounds has in common a hydroxyaminosulfate group and a β-thioglucosyl residue attached to variable side chain, which characteristics depend on the precursor aminoacid and the number of ciclyzations [26,133]. These compounds are known to respond to different biotic or abiotic stress conditions [26] under stress-specific basis. In *Arabidopsis*, drought induced aliphatic glucosinolates and flavonoids but repressed accumulation of the phytoalexin camalexin, whereas soil waterlogging induced all kinds of secondary metabolites [134]. The actual function of the accumulation of flavonoids and other phenolics and glucosinolates on abiotic stress tolerance is not known yet.

#### 3.2.3. Carotenoids and Other Terpenoid Derivatives

Carotenoids and xanthophylls are lipophilic compounds synthesized in plants from isopentenyl pyrophosphate (IPP) via the plastidial methyl erythritol phosphate (MEP) pathway. The carotenoid pathway is very well established. After several rounds of addition in the MEP pathway, an intermediate named geranylgeranyl pyrophosphate (GGPP, C20) is generated from IPP. The first committed step of carotenoid biosynthesis is the condensation of two molecules of GGPP to form a colorless phytoene (C40). Then, the enzymes phytoene desaturase and carotene desaturase convert phytoene into lycopene via the intermediates phytofluene, carotene, and neurosporene. Then, lycopene is cyclized into γ-carotene, which is subsequently converted to β-carotene. In a two-step hydroxylation, β-carotene is converted zeaxanthin and sequentially to violaxanthin by epoxidation. Finally, an arrangement in one epoxy ring of violaxanthin to form an allenic bond forms neoxanthin [135], the precursor of ABA in plants. These metabolites and others like α-tocopherol exert a positive effect against heat stress through the stabilization of the lipid phase of the thylakoid membranes [122]. In addition, high irradiation and especially UV radiation, has an impact on the composition of this kind of protective compounds. However, the role of carotenoids could not be restricted to UV radiation protection under stress conditions. For example, the overexpression of phytoene synthase gene in transgenic tobacco plants improved osmotic and salt stress tolerance, but by channeling carotenoid flux to ABA biosynthesis which led to increased levels of this phytohormone [136]. In citrus fruits, carotenoid is highly influenced by average temperature and rainfall, being higher in fruits developed under Mediterranean conditions than under tropical climate [137]. In particular, citrus limonoids and particularly the triterpenoid limonin, which is assumed to be responsible of the delayed bitterness phenomenon, occurs in juice sacs of citrus as a result of physical damage or field freeze. The tasteless precursor limonin A-ring lactone is catalyzed into bitter limonin by limonin d-ring lactone hydrolase at pH 6.5 or lower [138].

#### 3.2.4. Secondary Metabolites, Stress Tolerance and Fruit Quality

In crop plants, preadaptation is also an important mechanism in abiotic stress tolerance. There are important trade-offs between stress adaptation and yield in crops depending on how well cultivars cope with changing environment. In drought tolerance, the most typical traits are overaccumulation of carbohydrates and aminoacids but also changes in phenylpropanoids leading to differential flavonoid profiles [139]. This is agreement with previous findings where higher concentrations of phenylpropanoids such as caffeoylquinic acid and phenylalanine were found in tolerant model plant genotypes whereas sinapic acid and flavonoids such as quercetin were higher in sensitive species [140]. To this respect, concentration of bioactive secondary metabolites such as flavonoids in edible parts of plants is also severely affected by stress conditions, altering health and organoleptic properties as well. In tomatoes, water stress has an influence in the chemical composition of fruits depending on the relative sensitivity or tolerance of plants. Fruits of the drought-sensitive cultivar “Josefina” showed a significant decrease in hydroxycinnamic acids and flavonoid glycosides in response to water deficit whereas tolerant “Zarina” did not show such a response. However, grafting of the sensitive cultivar on the tolerant one had a positive effect on metabolite content of fruits after stress treatment, indicating that this could be an efficient tool to improve crop quality even under water deprivation [120].

## 4. Development of Metabolic QTL for Improving Stress Tolerance

Gene expression is a complex process that is not only controlled by a specific promoter, there are many different trans factors and epigenetic mechanisms that influence final gene expression [20]. Therefore, the solely identification of genes (molecular markers) in a particular genetic background cannot ensure the performance of a given genotype or the occurrence of certain compounds. Almost every agronomical trait is controlled by an intricate network involving an unknown number of genes making phenotype variation in natural populations a quantitative trait. For this reason, in plant breeding programs oriented towards the improvement of stress tolerance it is necessary to implement quantitative trait mapping analysis strategies, this is the statistical association of genetic markers with phenotypic variation, thus defining quantitative trait loci or QTLs [141]. Using these strategies, many QTLs associated to yield and stress tolerance in crops have been identified [141]. In maize and rice, one of the most important crops worldwide, many efforts have been oriented towards the identification of QTLs that may aid in marker-assisted breeding of cultivars with improved yield under stressful conditions [142–145]. As mentioned above, soil flooding is an important stress factor that causes a severe reduction in shoot and root growth in maize, thus reducing the tilling capacity [142]. In a F2 population developed from a cross between two maize inbred lines (waterlogging tolerant × waterlogging sensitive) more than 25 QTLs with associated to the selected phenotype traits, showing the potential of this approach to select markers for future breeding strategies [142]. Another important stress factor involved in severe yield loss in maize is water limitation [143]. In a recent publication, QTLs associated to different phenotype traits were identified in a maize recombinant inbred line population. However, although QTL expression over the years within a given water regime was quite stable, when comparing different drought conditions this stability decreased drastically, suggesting a strong environmental pressure on the selected phenotype traits [143]. Other approaches, by combining previous knowledge, like in the *sub1* locus associated to submergence tolerance in rice [144] or *OzT3* and *OzT9* associated to the apparition of leaf symptoms under ozone exposure [145], have been followed to identify QTL markers associated to stress tolerance. In this latter, a multiplexed approach was used by combination of gene expression, enzyme activity and metabolite analyses pointing to the ROS scavenging and antioxidant turnover metabolism as an important tolerance trait under high ozone [145]. However, although all the results are promising it seems clear that phenotype traits are subjected to a strong environmental control reinforcing the need of integrative parameters.

The complex interaction between genotype and environment along with the fact that metabolites integrate these two components has favored an increasing tendency to use metabolites as selection markers in crop breeding programs [146]. Regarding this, most efforts have been oriented to cultivar selection, rootstocks and varieties with improved tolerance to yield- and quality-limiting stress factors.

**Figure 2:**
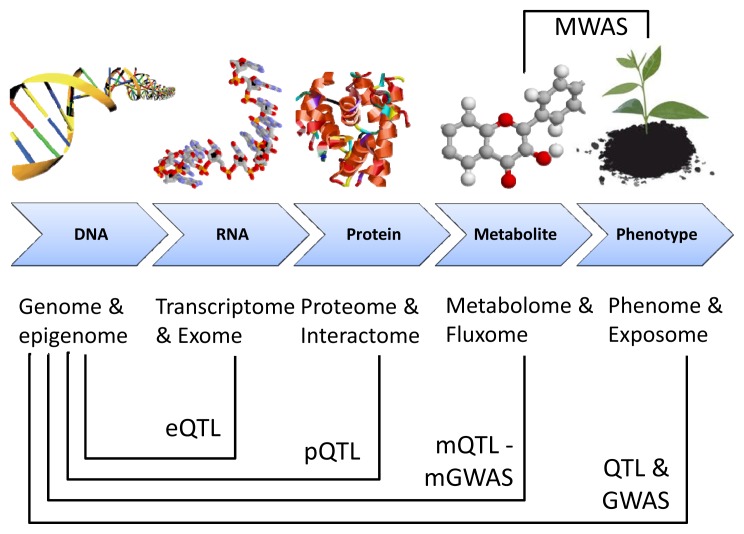
Genome mapping of molecular phenotypes. The levels of organization are depicted in the *x*-axis: from DNA to phenotype. Mapping of the molecular phenotypes onto the genome is achieved by quantitative trait loci (QTL) mapping and genome-wide association (GWAS) techniques. All profiling techniques but metabolome-wide association studies (MWAS) require genetic data. Adapted from [147].

The development of biomarkers is oriented towards the prediction of phenotypical properties before these features become apparent. This has been made possible by the development of metabotypes or the genetic determinants of metabolic phenotypes through metabotype quantitative trait locus (mQTL) mapping and metabolomic genome-wide association studies (mGWAS) in a rigorous statistical genetics framework, deriving associations between metabolite concentrations and genetic polymorphisms [147]. This was initiated with mapping of QTL for gene expression profiles, proteins and metabolites (Figure 2). The most important aspect is that metabolite biomarkers do not depend on genomic sequence availability and, as mentioned above, overcome the problem of complex and strongly environmentally-controlled traits [148].

A mQTL mapping consists in computing an association between genomic markers (SSR, SNPs or CNVs) and metabolic markers. However, mQTL mapping implies two problems: the identification of the candidate gene and the candidate metabolite. For these reasons, only quantitative metabotypes that are accurately defined are mapped allowing a more focused candidate gene search [147].

Preliminary studies aimed to analyze metabolite profiles of tomato interspecific introgression lines between wild *Solanum pennelli* and *Solanum lycopersicon* cv M82 and to map specific fruit metabolite fingerprints to whole-plant phenotypes [149]. Since then, other studies have attempted to map specific metabolite fingerprints to quantitative phenotype traits. Meyer and co-workers, using a rear inbred line population obtained from outcrossing C24 and Col-0 *Arabidopsis thaliana* accessions, found a specific metabolite signature associated to high biomass using canonical correlations [150]. Metabolites with negative correlation were fructose 6-phosphate, glucose 6-phosphate and citrate, suggesting a relationship with the inhibition of energetic metabolism: glycolysis and citric acid cycle; others with a positive correlation were nitrogenous compounds such as ornithine and polyamines putrescine and spermidine, suggesting an upregulation of cell division and nitrogen assimilation as key traits behind increasing biomass. As a follow up, using a similar combined approach it was possible to investigate the phenomenon of heterosis or hybrid vigor [151]. In crop plants such as corn, biomass production is an important agronomical trait that integrates both biosynthetic and catabolic activity. In a recent publication, by using the metabolite profiles of selected parental and hybrid corn lines it was possible to identify metabolic traits showing different modes of inheritance. In addition, the metabolite profiles between hybrid lines were more homogeneous than between the parental lines [152]. Acclimation to low temperature stress has been also a topic deeply examined from the perspective of developing a particular metabolic signature that could be associated to gene expression. Following a similar approach as described before, a population of nine *Arabidopsis* accessions acclimated to different environmental temperatures (representing habitats from 16° to 66° northern latitude) and subjected to cold stress it was found that particular transcript and metabolite profiles correlated with the ability to cold acclimate [153]. In addition, results indicated an overreduction of photosynthesis and hormonal regulation and an induction of photoprotective flavonoids. The stress tolerance phenotype, as well as other agronomical trait, is highly influenced by growing conditions that might mask to a great extent the desired phenotype. In addition, depending on the tissue monitored, these differences might be greater or just invalidate the biomarker chosen [112].

## 5. Conclusions and Future Prospects

The study of the metabolome represents the integration of the genetic background and the influence of the environmental conditions, thus describing more accurately the phenotype of a given plant species. In response to adverse abiotic stimuli, plants orchestrate an array of responses oriented to stress avoidance, defense or resistance, depending on the particular stress tolerance. Whereas stress avoidance involves modifications in growth habits and seasonal quiescence, defense and resistance are necessarily associated to strong metabolic modifications. Among all metabolic responses, alterations in the primary metabolism are the most evident and involve changes in levels of sugars and sugar alcohols, aminoacids and TCA cycle intermediates, showing general trends in response to abiotic stress. However, changes in the secondary metabolism are more specific of a given species and are highly specific of the particular stress condition. The integration of genome and metabolome for phenotype prediction is particularly interesting in crop breeding, since the selection based solely on genetic markers is strongly biased by the influence of the environment. The development of mQTL and MWAS markers for crop selection and improvement of abiotic stress tolerance in crops will help to overcome the problems derived from differing environmental conditions. This field will take advantage of the new plant genomes recently issued [154,155] and the modern and more powerful metabolite profiling tools.
